# ALDB: A Domestic-Animal Long Noncoding RNA Database

**DOI:** 10.1371/journal.pone.0124003

**Published:** 2015-04-08

**Authors:** Aimin Li, Junying Zhang, Zhongyin Zhou, Lei Wang, Yujuan Liu, Yajun Liu

**Affiliations:** 1 School of Computer Science and Technology, Xidian University, Xi'an, Shaanxi, PR China; 2 School of Computer Science and Engineering, Xi'an University of Technology, Xi'an, Shaanxi, PR China; 3 Department of Molecular and Cell Biology, School of Life Sciences, University of Science and Technology of China, Hefei, PR China; 4 State Key Laboratory of Genetic Resources and Evolution, Kunming Institute of Zoology, Chinese Academy of Sciences, Kunming, PR China; 5 Xi'an DongXing Branch, CMST Development Co. Ltd., Xi'an, Shaanxi, PR China; 6 Higher Technology College, Xi'an University of Technology, Xi'an, Shaanxi, PR China; National Center for Biotechnology Information, UNITED STATES

## Abstract

**Background:**

Long noncoding RNAs (lncRNAs) have attracted significant attention in recent years due to their important roles in many biological processes. Domestic animals constitute a unique resource for understanding the genetic basis of phenotypic variation and are ideal models relevant to diverse areas of biomedical research. With improving sequencing technologies, numerous domestic-animal lncRNAs are now available. Thus, there is an immediate need for a database resource that can assist researchers to store, organize, analyze and visualize domestic-animal lncRNAs.

**Results:**

The domestic-**a**nimal **l**ncRNA **d**ata**b**ase, named ALDB, is the first comprehensive database with a focus on the domestic-animal lncRNAs. It currently archives 12,103 pig intergenic lncRNAs (lincRNAs), 8,923 chicken lincRNAs and 8,250 cow lincRNAs. In addition to the annotations of lincRNAs, it offers related data that is not available yet in existing lncRNA databases (lncRNAdb and NONCODE), such as genome-wide expression profiles and animal quantitative trait loci (QTLs) of domestic animals. Moreover, a collection of interfaces and applications, such as the Basic Local Alignment Search Tool (BLAST), the Generic Genome Browser (GBrowse) and flexible search functionalities, are available to help users effectively explore, analyze and download data related to domestic-animal lncRNAs.

**Conclusions:**

ALDB enables the exploration and comparative analysis of lncRNAs in domestic animals. A user-friendly web interface, integrated information and tools make it valuable to researchers in their studies. ALDB is freely available from http://res.xaut.edu.cn/aldb/index.jsp.

## Introduction

Noncoding RNAs (ncRNAs) are a large class of RNAs that do not encode proteins. Based on their sequence characteristics and biological functions, ncRNAs can be classified as (1) house-keeping RNAs; (2) small ncRNAs; (3) long noncoding RNAs (lncRNAs). As the advances of sequencing technologies, thousands of lncRNAs have been identified in animals and plants [1–3]. Functional research has shown that lncRNAs play important roles in fundamental biological processes such as dosage compensation, transcriptional regulation and epigenetic regulation [4–6]. To identify lncRNAs cleanly, and to simplify their analysis by avoiding the complications arising from overlap with other types of genes, we here focus on intergenic long noncoding RNAs (lincRNAs), a family of lncRNAs that are located within intergenic regions.

Domestic animals show considerable phenotype diversity due to thousands of years’ domestication. Identification of domestic animal lincRNAs has been previously conducted in the gonad transcriptome of two extreme male pigs, chicken skeletal muscle and bovine skin using RNA-seq data [7–10], and also been performed in bovine using EST data [11] (Table A in S1 File). These reports indicate that some lincRNAs present homology with human, and suggest that some lincRNAs serve important roles in muscle development or in the modulation of pigmentation processes [7–10]. Recently, Zhou *et al*. [12] have also carried out the genome-wide identification of pig lincRNAs from RNA-seq and NCBI UniGene data using a computational pipeline with a stringent criterion. Among the lincRNAs identified by them, *linc-sscg2561* exhibits synteny and sequence conservation. Both *linc-sscg2561* and its neighbor *Dnmt3a*, associated with emotional behaviors, show differential expression in the frontal cortex between domesticated pigs and wild boars, suggesting *linc-sscg2561*’s possible role in pig domestication. In this study, we used RNA-seq transcriptome datasets in combination with NCBI UniGene data to identify the lincRNAs of chicken and cow employing a similar method as Zhou *et al*.’s [12]. We built a database, named ALDB, to store and explore lncRNAs.

Compared with existing lncRNA databases, such as NONCODE and lncRNAdb [13,14], ALDB has several distinctive features (Table 1). ALDB provides the expression levels of lncRNA and mRNA genes from various tissues for domestic animals. As lncRNAs are known to possibly interact with chromatin proteins to positively or negatively regulate the expression of neighboring genes [15–17], we also presented the expression of ten protein-coding genes adjacent to lincRNAs. QTLs are important to analyzing putative functions of genes. In ALDB, users can find the lincRNAs that are located in QTL regions. In summary, ALDB provides series of functions to facilitate research on domestic animal lncRNAs.

**Table 1:** Comparison between ALDB and related databases.

Database	lncRNA*	Expression	QTL	Source	Species	Description	Ref
NONCODE	16188**	Human, mouse	×	Literature	10 species	An integrated knowledge database of non-coding RNAs; http://www.noncode.org/	[13]
lncRNAdb	16	Human, mouse	×	Literature	Around 60 species	A reference database for long noncoding RNAs; http://www.lncrnadb.org/	[14]
PLncDB	0	Arabidopsis	×	Tiling Arrays, RNA-seq	Arabidopsis	A plant long noncoding RNA database; http://chualab.rockefeller.edu/gbrowse2/homepage.html	[18]
NRED	0	Human, mouse	×	Design arrays	Human, mouse	A database of long noncoding RNA expression; http://nred.matticklab.com/cgi-bin/ncrnadb.pl	[19]
LncRNADisease	0	×	×	Literature	Human	A database for long-non-coding RNA-associated diseases;http://www.cuilab.cn/lncrnadisease	[20]
LNCipedia	0	×	×	Literature	Human	A comprehensive compendium of human long non-coding RNAs; http://www.lncipedia.org/	[21]
The Functional lncRNA Database	0	×	×	Literature	Human, mouse, rat	A repository of mammalian long non-protein-coding transcripts that have been experimentally shown to be both non-coding and functional; http://www.valadkhanlab.org/database	[22]
ChIPBase	0	Human	×	ChIP-Seq	6 species	A database for decoding the transcriptional regulation of long non-coding RNA and microRNA genes from ChIP-Seq data; http://deepbase.sysu.edu.cn/chipbase/	[23]
starBase	0	×	×	CLIP-Seq	Human, mouse, Caenorhabditis elegans	Decoding miRNA-ceRNA, miRNA-ncRNA and protein-RNA interaction networks from large-scale CLIP-Seq data; http://starbase.sysu.edu.cn/	[24]
ALDB	29276	Pig, chicken, cow	Pig, chicken, cow	RNA-seq, EST	Pig, chicken, cow	A domestic-animal long noncoding RNA database; http://res.xaut.edu.cn/aldb/	

* The number of domestic animal lncRNA transcripts.

** 16,188 chicken lncRNA transcripts were gathered from an unpublished source.

## Materials and Methods

### Ethics statement

This study involved analysis of existing, publicly available datasets, therefore no ethics statement is required for this work.

### Data acquisition and database content

ALDB currently provides the following data for further domestic-animal lncRNA research: (1) Annotated pig, chicken and cow lincRNAs; (2) Gene (including lincRNA and mRNA, etc.) expression levels in diverse tissue samples; (3) Overlap between lincRNA genes and QTLs; (4) Analysis and statistics on gene expression, QTLs and homology. For the convenience of analysis, we also integrated the Ensembl gene set [25].

#### Datasets used for lincRNA identification

The annotations of pig lincRNAs with CPC (Coding Potential Calculator) scores <0 were provided by Zhou *et al*. [12]. To identify a more comprehensive set of lincRNAs for chicken and cow, we jointly used both RNA-seq and UniGene (EST) datasets. EST sequences have been proven to be extremely valuable for the identification of lincRNAs in plants and mammals [26,27]. To minimize transcriptional noise, high quality and non-redundancy sequences (UniGene) were used in our study. We collected RNA-seq transcriptome datasets across 4 different experiments from the Sequence Read Archive (SRA)[28–31], and obtained chicken and cow unigenes from the NCBI UniGene database [32] (Tables B, C and D in S1 File).

Ensembl gene set (release 73) and the following genomic sequences, used for alignment, assembly and filtering, were downloaded from the Ensembl Genome Browser [25]: GG4.0 (chicken), UMD3.1 (cow).

#### Computational pipeline for identifying lincRNAs

LncRNAs can be crudely classified based on their genomic proximity to protein-coding genes, including sense, antisense, bidirectional, intronic or intergenic [33]. To identify lncRNAs neatly by escaping the complications arising from overlap with other types of genes, we currently focus on intergenic lncRNAs (lincRNAs), which lie within the genomic interval between two genes.

In this study, the bioinformatics pipeline of identification of lincRNAs was similar to Zhou *et al*.’s except that the cutoff of CPC scores was 0 (in this study), not -1 (in Zhou *et al*.’s study), in order to identify a more thorough lincRNA set [12,34].

**Fig 1:**
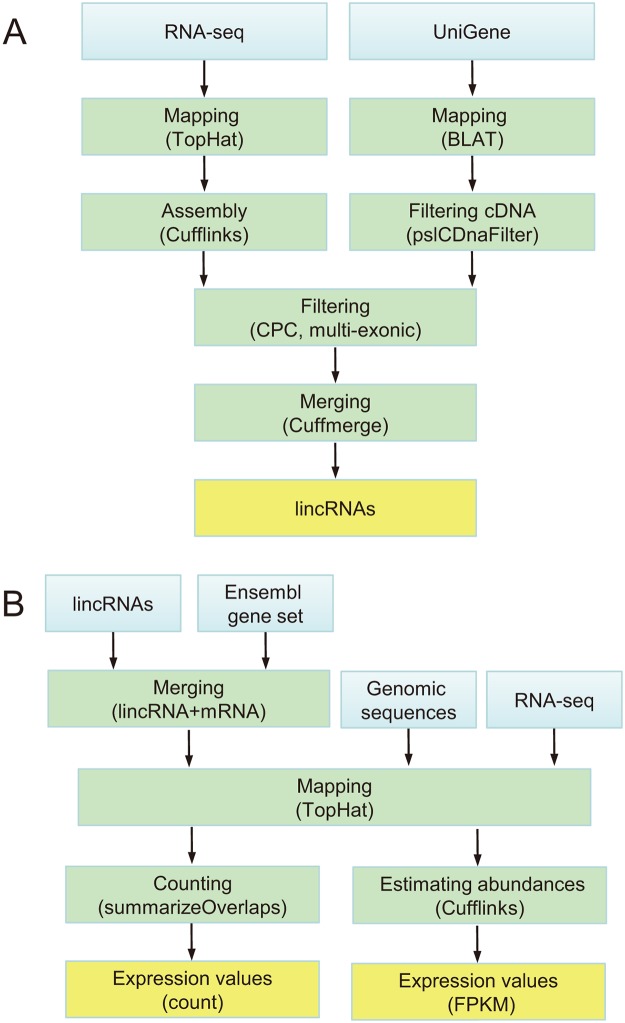
Identification of lincRNAs and calculation of expression levels. (**A**) Computational pipeline to identifying lincRNAs. (**B**) Pipeline of calculation of expression levels for protein-coding and lncRNA genes. Blue box: input data. Green box: processing. Orange box: outcomes.

Fig 1A highlights the main steps of the pipeline. The approach to extracting high-confidential and reliable transcripts from RNA-seq data differs from that from UniGene data.

For the RNA-seq data, all reads from each dataset were mapped to genomic sequences with an alignment tool TopHat (v2.0.8) [35] (where we set the option of searching novel splice variants be on and we provided gene annotations from Ensembl gene set to maximize spliced alignment accuracy). The resulting aligned data were then separately fed to an assembler Cufflinks (v2.0.2) [35] for their assembly to transcripts and the subsequent transcripts were compared with the Ensembl gene set (release 73) by using the Cuffcompare program in Cufflinks suite (v2.0.2) for the identification of novel transcripts (*class code = ”u”*). To reduce transcriptional noise, only those assembled transcript isoforms that had at least 3 reads to support their junctions and whose coverage of exon nucleotides was >80% in at least one sample were retained for further analysis [12].

For the UniGene data, each unigene was first mapped to the genomic sequences using BLAT (the BLAST-like alignment tool) (v35) [36], and then low-quality contigs were removed using the pslCDnaFilter program (http://hgdownload.cse.ucsc.edu/admin/exe/) with the parameters:-*minId = 0*.*95*, *-minCover = 0*.*25*, *-globalNearBest = 0*.*0025*, *-minQSize = 20*, *-minNonRepSize = 16*, *-ignoreNs-bestOverlap*. Contigs that equally mapped to multiple locations of the genomes were removed from the further analysis.

The resulting transcripts supported by UniGene and/or RNA-seq were further filtered as follows: (1) Size selection. The length of a transcript is at least 200 nt. (2) Keeping intergenic lncRNAs. The location of a transcript is at least 500 bp away from the closest annotated genes (Ensembl genes). (3) Coding potential test. The CPC score of a transcript is less than 0 [34]. (4) Retaining multi-exonic transcripts. A transcript has at least 2 exons [12].

Finally, the retained confidential high-quality transcripts were combined by using the Cuffmerge (v2.0.2) program in Cufflinks suite with default parameters to form final non-redundant lincRNA catalog (We used Cuffmerge as follows: *cuffmerge-s genome_file-o directory_of_output_file input_list_file*).

Using the above pipeline, we identified 8,923 chicken lincRNA transcripts from 6,151 loci, and 8,250 cow lincRNA transcripts from 6,213 loci, respectively.

#### Characterization of expression profiles

In order to profile the expression patterns of the lincRNAs and other Ensembl genes, we derived count and FPKM (fragments per kilobase per million mapped reads) expression levels from RNA-seq data by using TopHat, Cufflinks and summarizeOverlaps [37]. Of the 13 RNA-seq datasets from the SRA and EBI databases (Table C in S1 File), we selected the following RNA-seq datasets which were produced from relatively higher-depth sequencing and contained more tissue samples: ERA178851 (pig, 20 samples, 10 tissues), SRA059960 (chicken, 26 samples, 9 tissues; cow, 27 samples, 9 tissues).

The following approach begins with lincRNAs that we identified, and concludes with gene expression levels (Fig 1B). First, lincRNAs and Ensembl genes were combined to form a novel annotated gene set. Then, RNA-seq reads for each sample were mapped to the genomic sequences with TopHat (with the option—*no-novel-juncs* to skips gene and transcript discovery), and the novel annotated gene set were also fed to TopHat (with the option-*G*). The resulting alignment files were then fed to Cufflinks to quantify gene abundance with the parameter-*G* and output expression values into the file *genes*.*fpkm_tracking*. For achieving count expression values, we used the summarizeOverlaps function from the GenomicRanges package (version 1.14.4) [37] with the default parameter *Mode = Union*. Reads were counted from a list of Binary Alignment/Map (BAM) files derived from TopHat and count expression values were returned in a matrix file for possible use in further analysis such as those offered in DESeq and edgeR [38,39].

#### Integration of animal QTLs

QTLs provide useful information to link genome loci with phenotypes [40]. We downloaded 9,862 pig QTLs, 3,919 chicken QTLs and 8,305 cow QTLs from the animal QTL database (release 22) [41]. These QTLs and their overlap with lincRNAs are curated in ALDB and can be conveniently visualized in GBrowse.

### Database construction

#### Database design and implementation

The web pages of ALDB were built using a combination of Java Server Pages (JSP) and jQuery EasyUI 1.3.5 (http://www.jeasyui.com). They run on a Linux system (Centos 6.4) and are powered by a Tomcat (7.0.53) server. All data were stored in a MySQL (5.1.66) database (http://www.mysql.com), except that the lincRNA and expression data for downloads were stored as files in multiple format (GTF, GFF3, FASTA, etc.).

We created nine MySQL tables for ALDB, and the relationship of these tables is presented in S2 File. The description of these tables is listed in Table 2.

**Table 2:** Description of MySQL database tables.

Table	Description
*gene*	The lncRNA genes and the Ensembl genes.
*transcript*	The lncRNA transcripts and the Ensembl transcripts.
*expression*	The expression levels of all genes.
*neighborgenes*	The neighboring genes of each gene.
*neighborlnc*	The neighboring lncRNA genes of each protein-coding gene.
*qtl*	The QTL data.
*overlapqtl*	The overlap between genes/transcripts and QTLs.
*exon*	The start and end locations of each exon.
*sequence*	The nucleotide sequence of each transcript.

#### Generic genome browser

We used GBrowse 2.55 [42], a web-based application for displaying genomic annotations and other features, to visualize the genomes, lincRNAs, Ensembl gene set (release 73) and Animal QTLs (release 22). Gbrowse was built by Perl CGI scripts and was powered by an Apache server (2.2.15). In Gbrowse, users can scroll and zoom through arbitrary regions of a genome, enter a region of the genome by searching for a landmark or performing a full text search of all features, and enable and disable tracks. Users can also download GFF3/FASTA files of derived features from GBrowse.

#### Online BLAST service

To enable sequence homology searches against lincRNAs, we incorporated a standalone BLAST server using NCBI's wwwblast tool [43] (version 2.2.26, http://www.ncbi.nlm.nih.gov/staff/tao/URLAPI/wwwblast/), which can be easily accessed through web browsers.

## Results

### Web interface of ALDB

**Fig 2:**
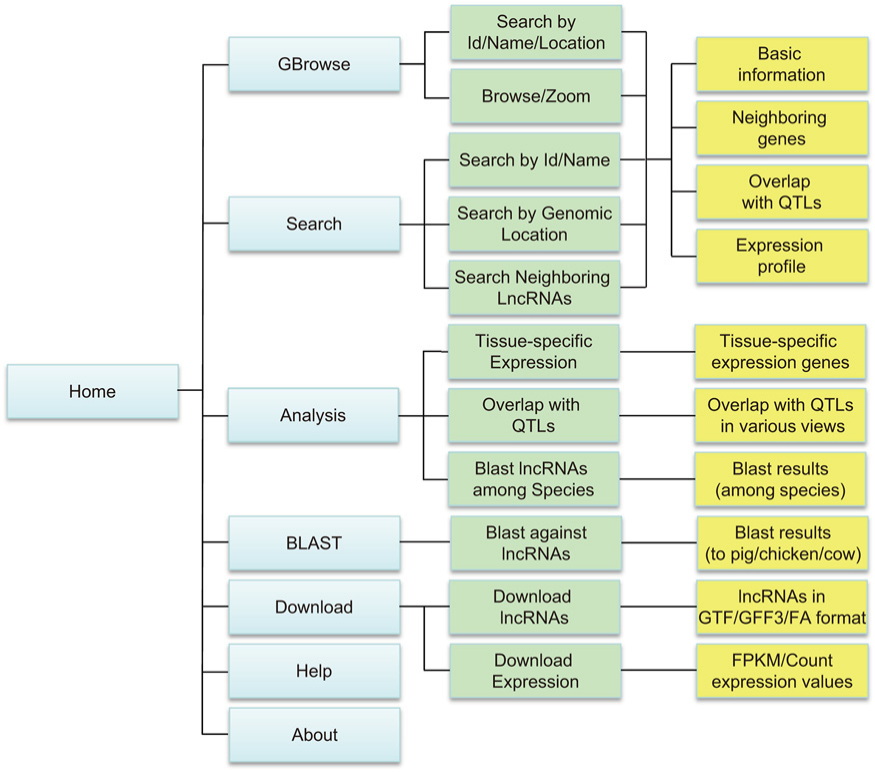
Schematic illustration of ALDB sitemap. Blue box: navigation bar. Green box: processing. Orange box: outcomes.

We created a freely accessible web interface for visiting ALDB. It comprises six core-page sections: “Home”, “GBrowse”, “Search”, “BLAST”, “Analysis” and “Download” (Fig 2).

#### Home page

Users can access ALDB at http://res.xaut.edu.cn/aldb/index.jsp. The home page contains an introduction to ALDB and provides a sitemap (Fig 2), which gives users an outline of ALDB and is convenient for them to view and retrieve the information that they are interested in.

#### GBrowse

**Fig 3:**
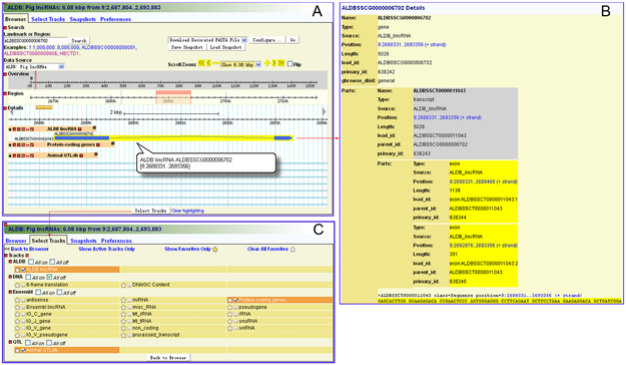
Visualization of genome annotation using GBrowse. (**A**) Users can search and browse features using GBrowse. (**B**) Detail page of a gene. (**C**) Tracks in GBrowse.

GBrowse greatly facilitates users to browse any region of interest in pig, chicken and cow genomes. It allows for a comprehensive analysis of the genomic neighborhood of a single gene or transcript. Users can select data source, and search transcripts or genes by their ids or names, or by a genomic region. A variety of track features can be accessed depending on the region’s position on the genomes, including lncRNA genes, protein-coding genes and QTLs (Fig 3A). In viewing the features, users can zooms out or zooms in a region to view the outline or details of a feature. If the user holds the cursor over a specified gene or transcript, a popup balloon will appear to show gene tracks, symbols and location information. Alternatively, by clicking on a specific gene, users can jump to a new corresponding gene information page to view its more considerable details (Fig 3B). Users can view different elements on different tracks by customizing tracks on the page “Select Tracks” (Fig 3C).

#### Search

Search functionalities are designed to facilitate users to retrieve useful information, such as the basic information of transcripts and genes, expression profiles, neighboring genes, overlap with QTLs and the lncRNA genes adjacent to a particular protein-coding gene. These functional units are contained in the “Search” section on the main navigation bar. This section mainly includes “Search by Id/Name”, “Search by Genomic Location”, and “Search Neighboring LncRNAs” pages.

**Fig 4:**
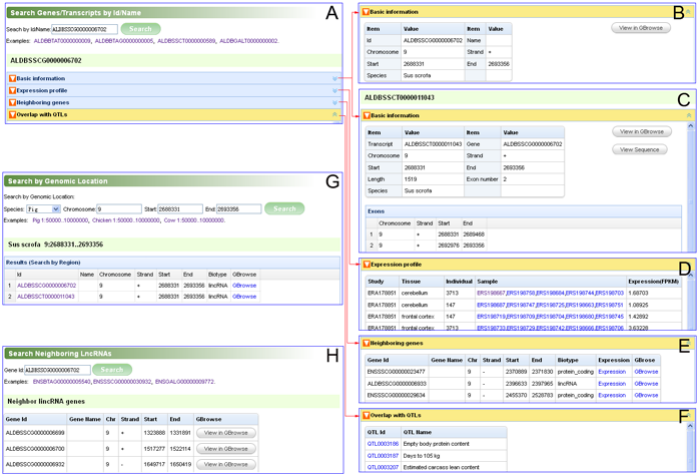
Screenshot of the search pages. (**A**) Search for transcripts or genes by their ids or names. (**B**) Basic information for a gene. (**C**) Basic information for a transcript. (**D**) The expression levels of genes in various tissue samples. (**E**) About ten genes adjacent to the current gene are represented. (**F**) The QTLs which overlap with the current gene or transcript. (**G**) Search for transcripts or genes by genomic region. (**H**) Search for neighboring lncRNAs of protein-coding genes.

In the “Search by Id/Name” page, users can find transcripts and genes by inputting their ids or names (Fig 4A). For a transcript or gene, the following relative information will be represented: “Basic information” (Fig 4B and 4C), “Expression profile” (Fig 4D), “Neighboring genes” (Fig 4E), “Overlap with QTLs” (Fig 4F). The content panels can be expanded or collapsed when their head titles (inverted triangle icons) are clicked.

The “Search by Genomic Location” page is designed to help users to retrieve all the genes and transcripts located in a specific genomic region (Fig 4G). If users are interested in a transcript or gene, they can click its id in the first column to view its details, or click “GBrowse” in the last column to visualize it in GBrowse.

The “Search Neighboring LncRNAs” page can be used to search the lncRNA genes adjacent to a protein-coding gene (Fig 4H).

#### BLAST

**Fig 5:**
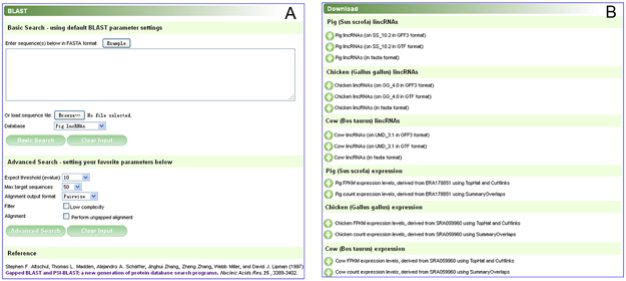
Screenshot of the BLAST and download pages. **(A)** The BLAST page. **(B)** The download page.

Since ALDB maintained nucleotide sequence data of lincRNAs, a sequence alignment tool, called wwwblast, based on the Basic Local Alignment Search Tool (BLAST) [43] was integrated into ALDB. Users can compare a query sequence with a library of lincRNA sequences using a BLAST search, and identify lincRNA sequences that closely resemble the query sequence. BLAST will meet users’ requirements for finding homologous transcripts or genes of interest. On the BLAST page (Fig 5A), users can supply one or more query sequences by uploading or directly pasting them to search against the available databases using BLAST’s default parameters. Users can also specify more parameters for a BLAST search to control its search sensitivity and result format, etc. The BLAST results will be displayed in another page in a pairwise format by default.

#### Analysis

Tissue-specific expression of genes tend to provide evidence for their function roles[44]. Users can find tissue-specifically expressed genes in the “Tissue-specific Expression” page, which were derived by using DESeq2 with the parameter *padj<0*.*1* according to their expression across various tissue samples.

The associations between lincRNAs and QTLs are presented in several forms: in trait hierarchies, by genes, by traits and by QTLs. These pages provide users with various information from different perspectives.

We blasted lncRNAs against one another among five species: pig, chicken, cow, human and mouse. Those transcripts with identical sequences and genomic locations were marked as “Identical” entries, and those with similar sequence information (query coverage>80%, subject coverage>80%, identity>0.8, e-value<1e-10) as “Similar” entries.

#### Downloads

The data of ALDB can be downloaded to perform local analysis. In the download page, users can download lincRNAs in FASTA, GFF3 and GTF formats. Data for downloads are divided per species and per content type, with information provided for the assembly of the species (Fig 5B).

We also provide the expression levels of lincRNA and Ensembl genes for downloads, which we derived from the datasets ERA178851 and SRA059960. The FPKM expression levels were obtained by TopHat (with the parameter—*no-novel-juncs*) and Cufflinks (with the parameter-*G*). The count expression levels were generated by summaryOverlaps with the parameter *Mode = "Union"*.

An up-to-date algorithm CNCI (Coding-Non-Coding Index) [45] is effective to differentiate coding and non-coding transcripts. For all the lincRNA transcripts archived in ALDB, we provided their CNCI scores [45] on the “Download” page of ALDB. Users can download these data and perform further analysis of lincRNA transcripts.

### Case study

In Zhou *et al*.’s study [12], they found 30 pig lincRNA genes significant differentially expressed between the brains of domesticated pigs and wild boars. Of those genes, we arbitrarily take *linc-sscg4116* (chr9:2688331–2693356) as an example to demonstrate the usage of ALDB.

First, find a gene identical to or related to *linc-sscg4116* in ALDB. Get the genomic location of *linc-sscg4116* from the GTF file which is released by Zhou *et al*. [12]. In the “Search by Genomic Location” page, select the “Pig” option and enter the chromosome, start and end locations of *linc-sscg4116*. After clicking the “Search” button, the result page displays a gene (*ALDBSSCG0000006702*) and a transcript (*ALDBSSCT0000011043*) (Fig 4G). Click “*ALDBSSCT0000011043*” and its details will be displayed (Fig 4C). Comparing *linc-sscg4116* with these details, we find they are identical. Therefore, *linc-sscg4116* is same as *ALDBSSCG0000006702* in ALDB.

Second, in the pig database of GBrowse, enter “*ALDBSSCT0000011043*” in the “Landmark or Region” text box and click the search button (Fig 3A). Neighboring transcripts, genes and overlapped QTLs can be visualized. Click *ALDBSSCT0000011043*, and the details of *ALDBSSCT0000011043* will be displayed in a new window (Fig 3B). In addition, the nucleotide sequence of *ALDBSSCT0000011043* and its genomic structure are available in this window (Fig 3B).

Third, in the “Search by Id/Name” page, enter *ALDBSSCT0000011043* and click the search button. The basic information of the transcript *ALDBSSCT0000011043* and overlap with QTLs will be presented (Fig 4C). Search for the gene *ALDBSSCG0000006702*, its expression levels in 10 tissues of two individuals can be available in the “Expression profile” panel (Fig 4D). Also, its neighboring genes will be displayed in the “Neighboring genes” panel (Fig 4E).

Fourth, in the “BLAST” page, enter the nucleotide sequence in the big text box and select “Cow lncRNAs” or “Chicken lncRNAs” in the database drop box (Fig 5A). After clicking the “Basic Search” button, we can check if it is homogeneous with cow or chicken lncRNAs.

Finally, in the “Download” page, the FASTA, GFF3 and GTF files of lincRNAs can be downloaded (Fig 5B). We can extract genomic structure and nucleotide sequences from these files. Also, we can download expression measures in count or FPKM format. These expression data can be supplied to DESeq, edgeR and so on [38,39], to identify differentially expressed genes, or can be used for co-expression analysis.

## Discussion

LincRNA genes have been identified to be associated with domestication and diseases [12,46]. Pig, chicken and cow are important domesticated animals and animal model in medical research. Therefore, it is necessary to identify lincRNA genes of these animals and construct a database to store them. To control the quality of transcripts, we integrated RNA-seq datasets and EST (UniGene) with a strict strategy to identify lincRNA genes. In pig, chicken and cow, we found 7,381, 6,151 and 6,213 lincRNA genes, respectively. To facilitate the study of these lincRNA genes, we built a database to store them and integrated expression levels and QTLs. Previous reports indicate that numerous lincRNA genes show significant tissue preferable expression and possibly interaction with chromatin proteins to positively or negatively regulate the expression of neighboring genes [15–17]. Thus, we presented tissue-specific genes, the expression levels of each lincRNA gene and its ten neighboring protein-coding genes in various tissues for pig, chicken and cow. These will help the function research of lincRNA genes in domesticated animals and the understanding of the domestication of pig, chicken and cow. Animal QTL database stores thousands of QTLs that are associated with the phenotypes of pig, chicken and cow. In this study, we found that most lincRNA genes were located in QTLs. Zhou *et al*. proposed that lincRNAs may contribute to the change of behavior, adipogenesis and muscle development in pigs [12] (Zhou *et al*. unpublished). Thus, to facilitate the trait research in lincRNA genes of domesticated animals, we integrated QTL datasets into ALDB. Users can conduct the analysis of lincRNAs’ overlap with QTLs and visualize the QTL datasets in GBrowse. Users can also integrate the expression of lincRNA genes, the expression of their neighbor protein-coding genes and QTLs to help the function research in domesticated animals. From the above, we can conclude that ALDB is a useful database for the function research of lincRNA genes in domesticated animals.

## Conclusions

The importance of domestic animals is unquestionable due to their global significance for agriculture and their particular relevance to biomedical research [47,48]. Recent reports suggest that lincRNAs have been implicated in muscle development, the modulation of pigmentation processes and domestication of domestic animals [7–10]. In this study, we constructed a unique and comprehensive database of domestic-animal lncRNAs, named ALDB, which currently embraces thousands of domestic-animal lincRNAs and other related data as well as several useful analysis tools.

12,103 pig lincRNAs, 8,923 chicken lincRNAs, and 8,250 cow lincRNAs were derived from a combination of RNA-seq and UniGene data. These lincRNAs were stored and well organized in ALDB. Domestic-animal lincRNA related data are also offered, such as expression levels and QTLs. Additionally, ALDB incorporates flexible search functionalities and integrates several analysis tools, such as BLAST and GBrowse.

In summary, ALDB provides a comprehensive ongoing platform specialized for domestic-animal lncRNA research. We hope that ALDB will become a valuable resource for the study of domestic animals, agriculture development and human diseases.

## Availability and Future Developments

ALDB is publicly available at http://res.xaut.edu.cn/aldb/index.jsp. ALDB supports all major latest web-browsers, preferably Mozilla Firefox, Google Chrome or Apple Safari for visualization and performance purposes. The ALDB will continue to grow with the further addition of data, analytical tools and other functionalities. The ALDB will be ongoing updated when novel lincRNAs of other domestic animals, for example, dog and horse are available. Currently, we identified and curated only intergenic lncRNAs. In future, we plan to incorporate other kinds of lncRNA datasets, such as intronic lncRNAs and natural antisense lncRNAs.

## Supporting Information

S1 File Datasets used in identification of domestic-animal lncRNAs. Table A—Data used in previous domestic-animal lncRNA research. Table B—Summary statistics for all RNA-seq data used in this study. Table C—RNA-seq data used in this study. Table D—UniGene datasets used in this study.
(PDF)

S2 File Entity-relation diagram of the ALDB database. (PDF)
