# Supplementary material for: ALDB: A Domestic-Animal Long Noncoding RNA Database
Source: PLoS One. 2015 Apr 8;10(4):e0124003. doi: 10.1371/journal.pone.0124003 (PMC4390226; doi:10.1371/journal.pone.0124003)
Supplement: S1 File — Table A—Data used in previous domestic-animal lncRNA research. Table B—Summary statistics for all RNA-seq data used in this study. Table C—RNA-seq data used in this study. Table D—UniGene datasets used in this study. (PDF) [file pone.0124003.s001.pdf]

Additional file 1: Datasets used in identification of domestic-animal lncRNAs

Table A - Data used in previous domestic-animal lncRNA research

Organism	Tissue	Number of Samples	Data	Description	Reference
Pig	gonad	2	RNA-seq	2,047 lincRNAs; unpublished	[1]
Chicken	skm	1	RNA-seq	281 lincRNAs; genomic structure unpublished	[2]
Bovine	skin	2	RNA-seq	4,365 lincRNAs; unpublished	[3]
Bovine	-	-	EST	23,060 ncRNAs;	[4]
Bovine	-	-	EST	449 lincRNAs;	[5]

Table B - Summary statistics for all RNA-seq data used in this study

Organism	Number of Samples	Number of Tissues	Tissue
Pig	93	19	Frontal cortex, occipital cortex, heart, spleen, cerebellum, hypothalamus, lung, liver, longissimus dorsi, kidney, abdominal fat, endometrium, sperm cell, colon, small intestine, lymph node, frontal lobe, placenta, testis
Chicken	36	10	Testes, spleen, skm, lung, liver, kidney, heart, colon, brain, ovary
Cow	27	9	Testes, spleen, skm, lung, liver, kidney, heart, colon, brain

Table C - RNA-seq data used in this study

Raw Data	Number of Samples	Tissue	Reference
ERA178851	20	Frontal cortex, occipital cortex, heart, spleen, cerebellum, hypothalamus, lung, liver, longissimus dorsi, kidney	[6]
ERA209456	10	Brain frontal cortex	[7]
SRA109352	10	Hypothalamus	[8]
SRA065859	4	Heart, liver, lung, kidney	[9]
SRA031272	6	Longissimus dorsi muscle, abdominal fat, liver	[10]
SRA065370	8	Endometrium	[11]
SRA092777	4	Sperm cell	[12]
SRA093372	8	Endometrium	[13]

SRA057367	3	Pool of 11 pig tissues (colon, kidney, hypothalamus, spleen, small intestine, lymph node, liver, lung, frontal lobe, cerebellum, placenta), placenta, testis	[14]
SRA066357	20	Liver, testis	[15]
SRA064905	4	Ovary, testis, brain	[16]
SRA059960	53	Testes, spleen, skm, lung, liver, kidney, heart, colon, brain	[17]
SRA059267	5	Brain, liver, kidney, heart, skeletal muscle	[18]

Table D - UniGene datasets used in this study

Organism	Build	#UniGene Entries	Release Date
Sus scrofa (pig)	build #42	50106	Jul 9 2012
Gallus gallus (chicken)	build #46	33850	Sep 26 2012
Bos taurus (cow)	build #101	45364	Jul 9, 2012

References

1. Esteve-Codina A, Kofler R, Palmieri N, Bussotti G, Notredame C, Perez-Enciso M. Exploring the gonad transcriptome of two extreme male pigs with RNA-seq. BMC genomics 2011;12: 552.
2. Li T, Wang S, Wu R, Zhou X, Zhu D, Zhang Y. Identification of long non-protein coding RNAs in chicken skeletal muscle using next generation sequencing. Genomics 2012;99: 292-298.
3. Weikard R, Hadlich F, Kuehn C. Identification of novel transcripts and noncoding RNAs in bovine skin by deep next generation sequencing. BMC Genomics 2013;14: 789.
4. Qu Z, Adelson DL. Bovine ncRNAs are abundant, primarily intergenic, conserved and associated with regulatory genes. PLoS One 2012;7: e42638.
5. Huang W, Long N, Khatib H. Genome-wide identification and initial characterization of bovine long non-coding RNAs from EST data. Anim Genet 2012;43: 674-682.
6. Farajzadeh L, Hornshøj H, Momeni J, Thomsen B, Larsen K, Hedegaard J, et al. Pairwise comparisons of ten porcine tissues identify differential transcriptional regulation at the gene, isoform, promoter and transcription start site level. Biochem Biophys Res Commun 2013;438: 346-352.
7. Albert FW, Somel M, Carneiro M, Aximu-Petri A, Halbwax M, Thalmann O, et al. A comparison of brain gene expression levels in domesticated and wild animals. PLoS genetics 2012;8: e1002962.
8. Pérez-Montarelo D, Madsen O, Alves E, Rodríguez MC, Folch JM, Noguera JL, et al. Identification of genes regulating growth and fatness traits in pig through hypothalamic transcriptome analysis. Physiol Genomics 2013.
9. Li M, Tian S, Jin L, Zhou G, Li Y, Zhang Y, et al. Genomic analyses identify distinct patterns of selection in domesticated pigs and Tibetan wild boars. Nat Genet 2013;45: 1431-1438.
10. Chen C, Ai H, Ren J, Li W, Li P, Qiao R, et al. A global view of porcine transcriptome in three tissues from a full-sib pair with extreme phenotypes in growth and fat deposition by paired-end RNA sequencing. BMC Genomics 2011;12: 448.
11. Samborski A, Graf A, Krebs S, Kessler B, Bauersachs S. Deep Sequencing of the Porcine Endometrial Transcriptome on Day 14 of Pregnancy 1. Biol Reprod 2013;88.
12. Bruggmann R, Jagannathan V, Braunschweig M. In Search of Epigenetic Marks in Testes and Sperm Cells of Differentially Fed Boars. PLoS One 2013;8: e78691.
13. Samborski A, Graf A, Krebs S, Kessler B, Reichenbach M, Reichenbach H-D, et al. Transcriptome Changes in the Porcine Endometrium During the Pre-attachment Phase. Biol Reprod 2013:

biolreprod. 113.112177.
14. Groenen MA, Archibald AL, Uenishi H, Tuggle CK, Takeuchi Y, Rothschild MF, et al. Analyses of pig genomes provide insight into porcine demography and evolution. Nature 2012;491: 393-398.
15. Gunawan A, Sahadevan S, Neuhoff C, Große-Brinkhaus C, Gad A, Frieden L, et al. RNA Deep Sequencing Reveals Novel Candidate Genes and Polymorphisms in Boar Testis and Liver Tissues with Divergent Androstenone Levels. PLoS One 2013;8: e63259.
16. Brawand D, Soumillon M, Necsulea A, Julien P, Csárdi G, Harrigan P, et al. The evolution of gene expression levels in mammalian organs. Nature 2011;478: 343-348.
17. Merkin J, Russell C, Chen P, Burge CB. Evolutionary dynamics of gene and isoform regulation in Mammalian tissues. Science 2012;338: 1593-1599.
18. Barbosa-Morais NL, Irimia M, Pan Q, Xiong HY, Gueroussov S, Lee LJ, et al. The evolutionary landscape of alternative splicing in vertebrate species. Science 2012;338: 1587-1593.
