# Supplementary figures and images for: ALDB: A Domestic-Animal Long Noncoding RNA Database
Source: PLoS One. 2015 Apr 8;10(4):e0124003. doi: 10.1371/journal.pone.0124003 (PMC4390226; doi:10.1371/journal.pone.0124003)

Additional file 2. Entity-Relation diagram of the ALDB database.

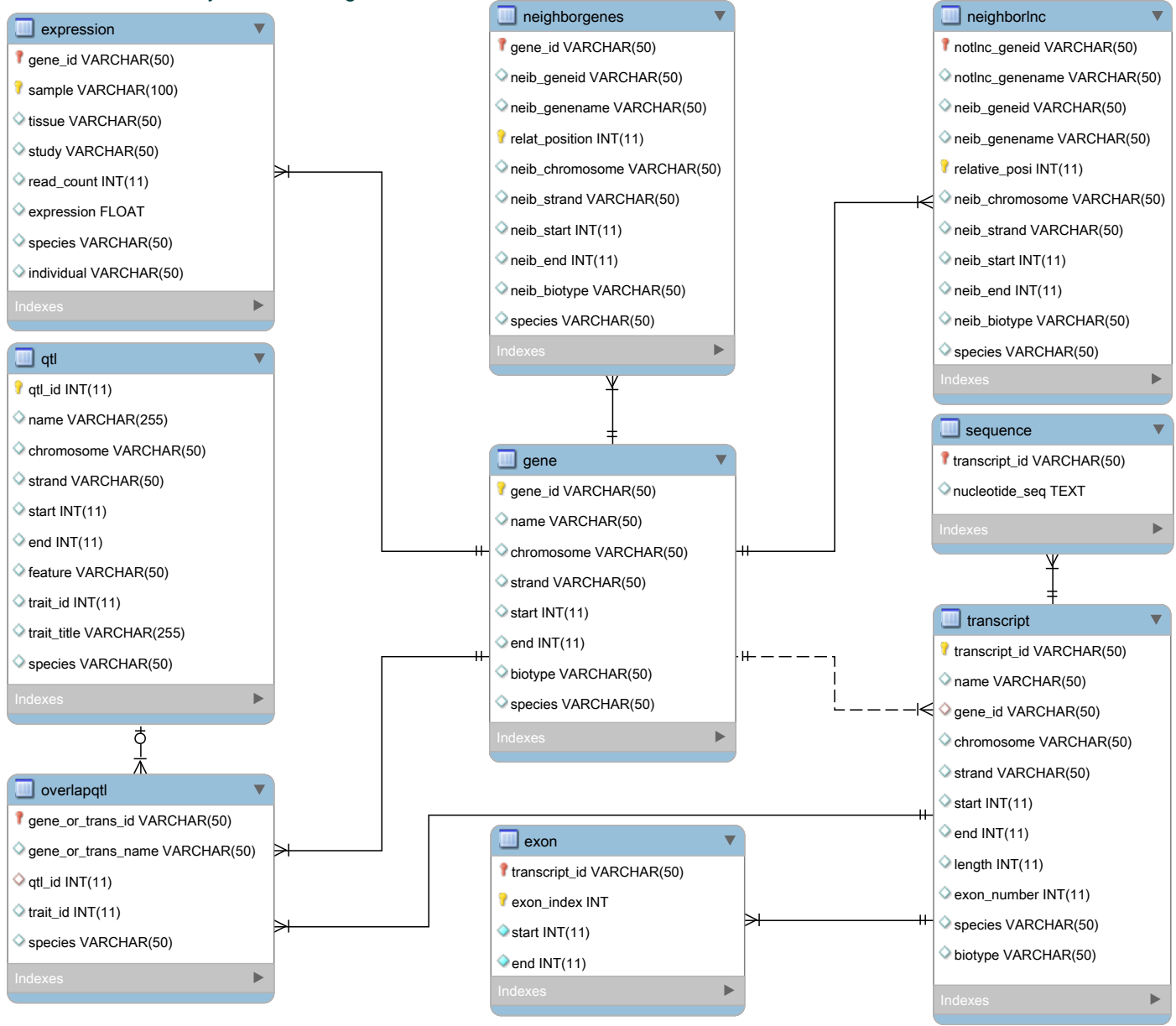

Supplement: S2 File — (PDF) [file pone.0124003.s002.pdf]
